# The Promotion of Research Progress of Zinc Manganate Cathode Materials for Zinc-Ion Batteries by Characterization and Analysis Technology

**DOI:** 10.3390/molecules28114459

**Published:** 2023-05-31

**Authors:** Xin Meng, Ziyi Cheng, Le Li

**Affiliations:** Shaanxi Key Laboratory of Industrial Automation, School of Mechanical Engineering, Shaanxi University of Technology, Hanzhong 723001, China

**Keywords:** zinc-ion batteries, cathode, ZnMn_2_O_4_, composite, defect

## Abstract

Zinc-ion batteries (ZIBs) have recently attracted great interest and are regarded as a promising energy storage device due to their low cost, environmental friendliness, and superior safety. However, the development of suitable Zn-ion intercalation cathode materials remains a great challenge, resulting in unsatisfactory ZIBs that cannot meet commercial demands. Considering that spinel-type LiMn_2_O_4_ has been shown to be a successful Li intercalation host, spinel-like ZnMn_2_O_4_ (ZMO) is expected to be a good candidate for ZIBs cathodes. This paper first introduces the zinc storage mechanism of ZMO and then reviews the promotion of research progress in improving the interlayer spacing, structural stability, and diffusivity of ZMO, including the introduction of different intercalated ions, introduction of defects, and design of different morphologies and in combination with other materials. The development status and future research directions of ZMO-based ZIBs characterization and analysis techniques are summarized.

## 1. Introduction

The rapid depletion of fossil fuels and their detrimental impact on the environment have led to a strong interest in nonpolluting renewable energy sources, such as solar, wind, and geothermal energy, which are becoming increasingly important to the vision of global sustainable development [1,2,3,4]. However, these renewable energy power sources are difficult to use directly due to their discontinuous, unstable, and uncontrollable characteristics [5,6]. An energy storage device (ESD) was created to regulate power output and enhance the grid’s resilience to renewable energy sources, thereby providing a reliable energy supply for both personal and industrial applications [7,8,9]. Among the existing EDSs, Li-ion batteries (LIBs) have received the most attention and have become the go-to option in the secondary battery market due to their high energy density [8,10,11,12,13]. The convenience of the commercialization of lithium salts cannot be overlooked, yet their reliance on limited and expensive lithium (and cobalt) resources and the potential fire hazards from organic electrolytes make them inadequate for large-scale usage [6,14,15].

**Figure 1:**
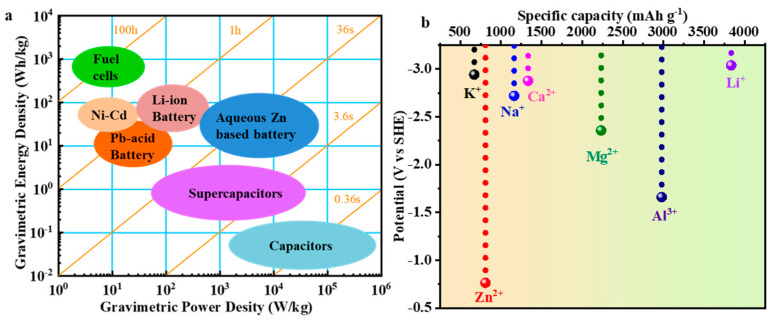
(**a**) Ragone plot of some electrochemical energy storage devices. (**b**) Schematic illustration of the working principle of zinc-ion battery. Reproduced with permission from [23], Copyright 2020, American Chemical Society. (**b**) Comparison of potential and specific capacitance of metal-ion (Li^+^, Na^+^, K^+^, Zn^2+^, Mg^2+^, and Al^3+^) batteries.

Recently, zinc-ion batteries (ZIBs) have been considered a potential candidate for large-scale energy storage applications in the post-LIB era due to the abundant reserves (75 parts per million in Earth’s crust), high theoretical capacity (819 mA·h·g^−1^), and low redox potential (−0.763 V vs. standard hydrogen electrode) of zinc, and the nonflammable and high ion conductivity (1 S·cm^−1^) of aqueous electrolytes (Figure 1) [6,16,17,18,19,20,21,22,23]. A ZIB device typically consists of four main components: an anode, a cathode, a separator, and an electrolyte. During the discharge process, Zinc ions are released from the anode, travel through the electrolyte, and eventually combine with the material of the cathode. Electrons traverse an external circuit, generating an external current from the cathode to the anode. Upon charging the battery, the system undergoes a reversal of the process and is restored to its pre-discharged condition. Researchers have formulated a range of materials that would satisfy the requirements of each component, based on the charging and discharging process. Cathode materials are an important research topic in the study of ZIBs. To date, various cathode materials, including vanadium-based materials [24,25], manganese-based materials [26,27], Prussian blue analogs [28,29], carbon-based materials [30,31], organic compounds [32,33], metal chalcogenides [34,35], MXene-based materials [36,37], and copper-based compounds [38,39], have been used as cathodes for ZIBs. Among them, zinc manganate (ZnMn_2_O_4_, abbreviated as ZMO), as a new type of bimetallic oxide with a spinel-like structure, is considered as a promising cathode material for aqueous ZIBs due to its abundant sources, large theoretical capacity, environmental friendliness, and high voltage. However, the low intrinsic conductivity, high volume change during charging and discharging, rapid capacity decrease, and inadequate rate performance of ZMO impede its further development. Many effective methods have been used to improve its electrochemical performance and reaction kinetics.

**Figure 2:**
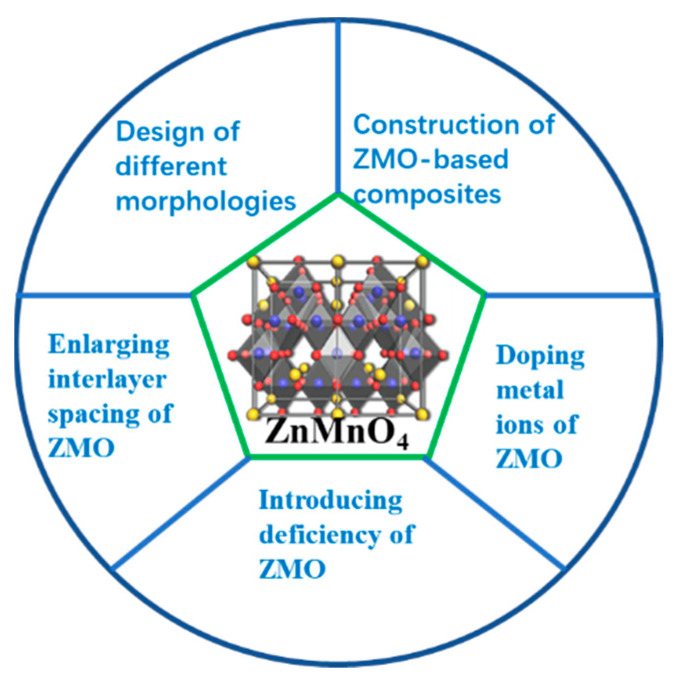
Summary of the research progress of zinc manganate materials.

Although reviews of manganese oxides as cathode materials for ZIBs continue to emerge, they mainly focus on synthesis, morphology design, and electrolyte optimization, rarely focusing on ZMO materials alone. The differences in the crystal structure and chemical composition of ZMO lead to differences in their energy storage mechanisms, which result in huge differences in electrochemical performance. This review first introduces the zinc storage mechanism of ZMO and then reviews the recent research progress in improving the interlayer spacing, structural stability, and diffusivity of ZMO, including the introduction of different intercalated ions, introduction of defects, and design of different morphologies and in combination with other materials (Figure 2). The development status and future research directions of ZMO-based ZIBs are summarized. This review expounds the electrochemical reaction mechanism of ZMO-based materials, which is helpful for the discovery of new ZMO-based cathode materials, provides a new perspective for the innovation of ZMO-based cathode materials, and promotes the rapid development and development of ZMO-based cathode materials in large-scale applications.

## 2. Zinc Storage Mechanism of ZMO

**Figure 3:**
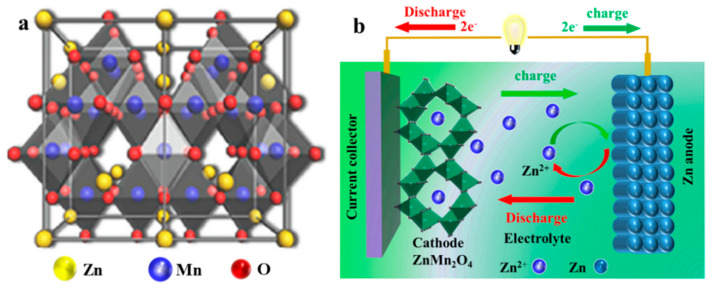
Schematic illustration of (**a**) ZMO crystal structure, and (**b**) the charge−discharge process of ZIBs based on ZMO cathode.

As a binary metal oxide, ZMO is a non-tunnel-type double-cation oxide spinel structure and belongs to the tetragonal crystal system, where Mn^3+^ occupies an octahedral position to form MnO_6_ groups, and Zn^2+^ ions take up tetrahedral positions to form ZnO_4_ groups (Figure 3a). The Jahn–Teller distortion effect is particularly pronounced in octahedral-coordinated Mn^3+^. Manganese and zinc in ZMO can act as buffer substrates for each other during charge–discharge cycling due to their different electrode potentials, making the charge–discharge cycling performance of ZMO superior to that of single-base manganese-based metal oxides [39]. Compared with monobasic manganese-based metal oxides, ZMO has higher specific capacity. This characteristic indicates that the synergistic effect of Zn and Mn can achieve a better energy storage effect. Consequently, ZMO has more prominent advantages in electrode material research and has the potential to become a new generation of ZIB electrode materials.

A typical ZMO-based ZIBs consists of a Zn anode, a ZMO cathode, and an electrolyte containing Zn^2+^. When the battery is charged, Zn^2+^ is gradually deintercalated from the tetrahedral site of spinel ZMO in a two-step process and dissolved in the electrolyte, releasing two electrons simultaneously. At the same time, zinc ions are deposited on the even surface of the zinc current collector, taking in two electrons from the external circuit. This condition is reversed during the discharge process (Figure 3b). Their overall response is as follows:

ZnMn_2_O_4_ ⇔ xZn^2+^ + 2xe^−^ + Zn_1−x_Mn_2_O_4_.

## 3. ZMO Modification Strategies by Characterization and Analysis Technology

ZMO is seen as a potential cathode material for ZIBs, but there are several difficulties that need to be overcome before it can be put into practical use. These challenges are as follows: (1) the dissolution of ZMO during cycling; (2) the formation of byproducts leading to the volume expansion of the cathode material and the deficiency of electrolyte; (3) the electrostatic repulsion between Zn^2+^ and ZMO crystals, affecting Zn^2+^. The intercalation/deintercalation and diffusion of ions are adversely affected. Therefore, researchers have developed various strategies, including designing different morphologies of ZMO, constructing ZMO-based composites, enlarging the interlayer spacing of ZMO, introducing oxygen deficiency of ZMO, and doping metal ions of ZMO, to overcome these challenges.

### 3.1. Design of Different Morphologies

In the past few years, various morphologies of ZMO, including hollow porous spheres, fibers, and rods, have been synthesized and applied to the cathode materials of ZIBs. Their morphology controls the physicochemical properties of cathode materials and determines their electron transport properties.

**Figure 4:**
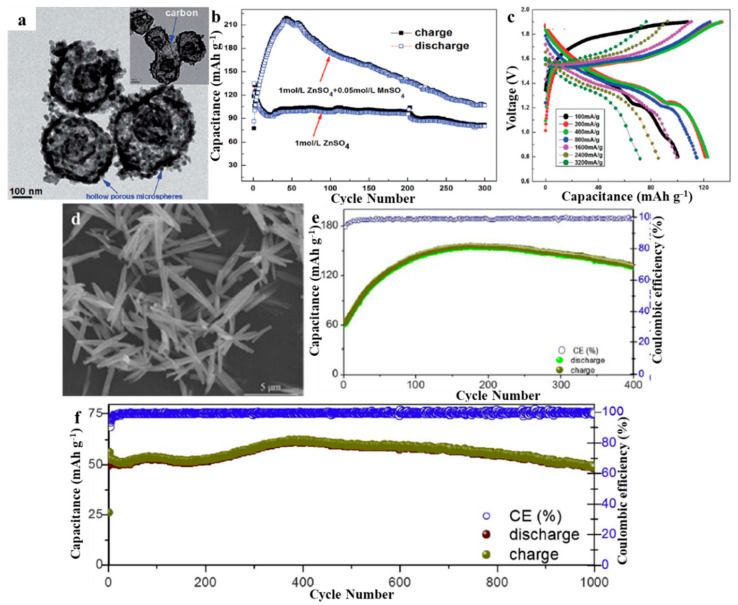
(**a**) TEM of ZMO. (**b**) The cycling performance of ZMO/Zn at 100 mA·g^−1^ with different electrolytes. (**c**) The rate performance of ZMO/Zn at different current densities based on 1 mol·L^−1^ of ZnSO_4_ with 0.05 mol·L^−1^ of MnSO_4_ in the range of 0.8−1.9 V. Reproduced with permission from [40], Copyright 2017, The Royal Society of Chemistry. (**d**) SEM of porous ZMO microrods. Cyclability curve for porous ZMO microrods at (**e**) 1 A·g^−1^ and (**f**) 2 A·g^−1^ rate. Reproduced with permission from [42], Copyright 2020, Elsevier.

Wu et al. synthesized hollow porous ZMO using a polyvinyl pyrrolidone-dispersed solvothermal carbon template method with subsequent annealing, and then used it as a cathode for ZIBs [40]. An experiment conducted using galvanostatic charge–discharge showed that, with the inclusion of 0.05 mol·L^−1^ of MnSO_4_ in the electrolyte, a reversible discharge capacity of 106.5 mA·h·g^−1^ at 0.1 A·g^−1^ could be maintained for 300 cycles with no capacity decay. It maintained a high capacity of 70.2 mA·h·g^−1^ at a high current density of 3.2 A·g^−1^ (Figure 4a–c). The hollow porous ZMO spinel structure, combined with the residual carbon distribution, creates a synergistic effect that prevents Mn dissolution during charging and discharging, resulting in its superior cycling and rate performance. Shama et al. synthesized ZMO using a one-step hydrothermal method with zinc aniline, acetate, and potassium permanganate as precursors and used it as a cathode for ZIBs [41]. The specific capacitance, energy density, and power density obtained from galvanostatic charge discharge were 50.02 F·g^−1^, 140.68 Wh·kg^−1^, and 5.633 kW·kg^−1^ respectively. The capacitance of the material was found to be 50 F·g^−1^, with a 99.26% retention rate after the 25th cycle. Soundharrajan et al. conducted a comparative study of the electrochemical activity of a ZMO microrod cathode produced via a straightforward coprecipitation process in a 0.1 M MnSO_4_ (MS) solution, as an additive in a 1 M ZnSO_4_ + 0.1 M MnSO_4_ (ZMS) electrolyte, and in a full-time 1 M ZnSO_4_ (ZS) electrolyte [42]. Utilizing super in situ X-ray diffraction (XRD), scanning electron microscopy (SEM), and transmission electron microscopy (TEM), systematic research has been conducted to explain the remarkable stability and reversibility of ZMO in ZMS electrolyte medium. The electrochemical equilibrium between Zn^2+^ and Mn^2+^ ions facilitates this excellent performance by stabilizing the Mn^2+^ additive in the Zn^2+^ (de)insertion in the electrolyte to reversibly electrodeposit/dissolve MnO_x_ on the cathode surface and reversibly insert Zn into the undissolved surface MnO_x_ layer (Figure 4d–f). Different morphologies affect the zinc storage properties of ZMO, and current research on ZMO morphologies mainly focuses on hollow porous spheres, fibers, and rods. Therefore, more morphologies of ZMO should be developed in future research to study its zinc storage mechanism.

### 3.2. Construction of ZMO-Based Composites

ZMO has large volume change, fast capacity attenuation, and poor rate performance during charging and discharging due to its inherent poor conductivity and slow reaction kinetics, which limits the practical application of ZMO in ZIBs. The electrochemical performance of the battery can be remarkably improved by the synergistic effect of complementary advantages among different materials. In this section, the research progress of ZMO composite with carbon-based materials, metal oxides, and MXenes is introduced.

#### 3.2.1. Composite with Carbon Materials

The storage performance of ZMO electrodes for zinc ions is significantly hindered by their low electrical conductivity and the considerable volume change that occurs when ions are inserted and removed. Therefore, the combination of ZMO and carbon-based materials in composite electrodes has been shown to be an effective way of enhancing conductivity and enabling faster electron transfer. The carbon composite provides the dispersion position for active materials, reduces self-accumulation and aggregation, acts as a buffer layer, and stabilizes the crystal structure of ZMO, which greatly improves its electrochemical performance.

**Figure 5:**
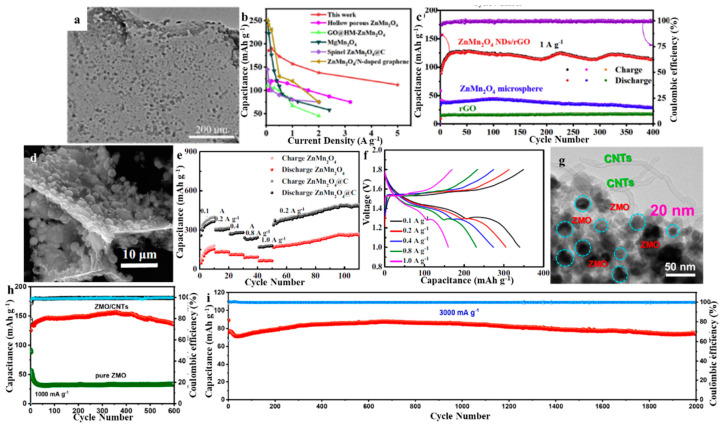
(**a**) TEM of ZMO NDs/rGO composite. (**b**) Comparison of the rate performance between this work and other reported cathode materials for ZIBs. (**c**) Long-term cycling stability of the ZMO NDs/rGO composite, pure ZMO microspheres, and rGO electrodes for 400 cycles at a high rate of 1 A·g^−1^. Reproduced with permission from [48], Copyright 2020, Elsevier. (**d**) SEM of ZMO@C composite. (**e**) The rate performance at different current densities and cycling performance of ZMO and ZMO@C. (**f**) Charge and discharge curves at different current densities. Reproduced with permission from [50], Copyright 2020, Elsevier. (**g**) TEM of ZMO/CNT composite. (**h**) Cycling performance at 1000 mA·g^−1^ of ZMO/CNTs and pure ZMO electrodes. (**i**) Long-life performance of ZMO/CNTs electrode at 3 A·g^−1^. Reproduced with permission from [55], Copyright 2022, Elsevier.

Graphene is a 2D form of carbon that is composed of sp^2^-hybridized carbon atoms arranged in a hexagonal honeycomb pattern. It is known for its excellent electrical conductivity and high electron mobility [43]. The graphene-containing nanocomposites form a conductive network and provide a buffer layer that effectively mitigates the volume expansion and self-aggregation of the active material, thereby enhancing cycle life and rate performance [8]. Chen et al. proposed the utilization of ZMO/N-doped graphene nanocomposite as the cathode material, which delivered a maximum discharge capacity of 221 mA·h·g^−1^ at 0.1 A·g^−1^ and a long cycle after 2500 cycles at 1 A·g^−1^ life of 97.4% [44]. The superior performance of this material is accredited to the combined effect of ultrafine ZMO nanoparticles (21 nm) and N-doped graphene dielectric. These components provide a rapid surface capacitive response, a minimal electron/ion transport path length, and a highly conductive dielectric. These features accelerate electron transport and fortify the composite structure to endure the volume increase during charging and discharging. Chen et al. prepared graphene-coated hollow ZMO microspheres (rGO@HM-ZMO) and used them as cathodes for ZIBs [45]. The addition of rGO increases the electronic conductivity and decreases the charge transfer resistance, while the hollow structure ameliorates the alteration of the crystal structure that occurs during successive Zn^2+^ insertion/extraction. The as-prepared rGO@HM-ZMO electrode demonstrated a remarkable specific discharge capacity of 146.9 mA·h·g^−1^ after 100 cycles with a current density of 0.3 A·g^−1^ and an impressive cycling stability with a capacity of 72.7 mA·h·g^−1^ without fading after 650 cycles at a higher current density of 1 A·g^−1^. Tao et al. synthesized nickel–cobalt co-substituted spinel ZMO nanoparticles uniformly loaded on N-doped rGO (ZnNi_x_Co_y_Mn_2−x−y_O_4_@N-rGO) using a one-step hydrothermal method and used them as a cathode for ZIBs [46]. The as-synthesized ZnNi_x_CoyMn_2−x−y_O_4_@N-rGO demonstrated remarkable electrochemical properties, with a reversible capacity of 95.4 mA·h·g^−1^ when tested at 1 A·g^−1^, and a capacity retention ratio of 79% after 900 cycles. As the current density increased from 0.01 A·g^−1^ to 1.5 A·g^−1^, a high capacity of 200.5 mA·h·g^−1^ to 93.5 mA·h·g^−1^ was attained. This good electrochemical performance is accredited to the co-substitution of nickel and cobalt elements, which is an effective way to promote Zn^2+^ deintercalation and stabilize the spinel structure to suppress the Jahn–Teller distortion of Mn^3+^. Qiu et al. designed novel nitrogen-doped ZMO oxygen-rich defects (N-ZnMn_2_O_4−x_) on 3D vertical graphene (VG) to form N-ZnMn_2_O_4−x_@VG core/shell arrays as cathodes for ZIBs [47]. Taking advantage of its high surface area, abundant active sites, swift electron/ion diffusion, and strong structural stability, the constructed device demonstrated a significant specific capacity (222 mA·h·g^−1^ at 0.1 A·g^−1^) and good rate performance (maintaining 61.58% from 0.1 to 3 A·g^−1^) along with remarkable cycle life (retaining 92.6% after 3000 cycles at 1.0 A·g^−1^). It offered an outstanding energy density of 278 W·h·kg^−1^ and an impressive power density of 3.62 kW·kg^−1^. The as-assembled ZIBs demonstrated the ability to be autonomously energized to a voltage of 1.5 V following oxidization in a natural atmosphere for a duration of 30 h and presented an impressive specific capacity of up to 176.8 mA·h·g^−1^ when tested at 0.2 A·g^−1^. Yao et al. synthesized composites (ZMO NDs/rGO) of ultrafine ZnMn_2_O_4_ nanodots uniformly anchored on rGO with strong interfacial interactions [48]. The as-synthesized ZMO NDs/rGO composite exhibited an excellent discharge capacity of 207.6 mA·h·g^−1^ at a current density of 0.2 A·g^−1^, as well as improved rate capability and long cycling stability. The as-assembled aqueous Zn//ZnMO NDs/rGO battery displayed a high energy density of 266 W·h·kg^−1^ at a power density of 137 W·kg^−1^ (Figure 5a–c). The material is rich in active sites, which shortens the ion diffusion path, improves the electrical conductivity, and results in good structural stability. Electrochemical analysis showed that the composites have good reaction kinetics, which is conducive to the efficient storage of zinc ions.

ZMO has been compounded with graphene, porous carbon polyhedrons, carbon networks, carbon nanfoam paper, carbon framework, porous carbon nanospheres, and carbon nanotubes to modify the zinc storage properties of ZMO. Yang et al. reported an in situ Raman study that dynamically probed the phase and structural evolution of spinel ZMO-based cathodes during the charge–discharge process, where spinel ZMO nanoparticles were immobilized on porous carbon polyhedrons (PCPs) [49]. Studies conducted in the field suggest that the electrochemical process is due to the reversible phase transition of spinel ZMO and λ-type MnO_2_ when Zn^2+^ is inserted or extracted, which is caused by the oxidation and reduction of Mn^3+^/Mn^4+^ and the efficient transportation of carriers. The as-prepared ZMO@PCP composite as a cathode exhibits extraordinary long-term cyclic stability with a capacity retention of 90.3% after 2000 cycles and a high reversible capacity of 125.6 mA·h·g^−1^ at a current density of 1 A·g^−1^. Wang et al. designed a novel egg waffle-like structure which comprised double-shelled ZnMn_2_O_4_ hollow microspheres embedded in a 2D carbon network (ZMO@C), and then used it as a cathode for ZIBs [50]. The ZMO@C electrode exhibits a capacity of 481 mA·h·g^−1^ at 0.2 A·g^−1^ after 110 cycles with good cycling stability (Figure 5d–f). The remarkable cycling stability of the ZMO@C electrode was due to the combined effect of the double-shelled ZMO hollow microspheres, which provide enough space to accommodate the volume change caused by the insertion/deinsertion of Zn^2+^, as well as the 2D continuous conductive interconnected carbon network, which facilitates the rapid electron transfer and ensures a strong structural integrity. Islam et al. prepared manganese-deficient ZMO@C (Mn-d-ZMO@C) nanostructured anode materials prepared using ZnO-MnO@C self-assembly for in situ generation of ZIBs [51]. Results from analytical methods supported the porous and crystalline nature of ZnO-MnO@C, as well as the in situ production of Mn-deficient ZMO@C. The Zn/Mn-d-ZMO@C cell exhibited a promising capacity of 194 mA·h·g^−1^ at a current density of 0.1 A·g^−1^, with 84% capacity maintained after 2000 cycles (at 3 A·g^−1^ rate). Additionally, 86% capacity retention was retained even after 1 year (150 cycles) at 0.1 A·g^−1^ current density. The performance improvement of this cathode stemmed mainly from the in situ orientation, porosity, and carbon coating. First-principles calculations confirmed the high electronic conductivity of the Mn-d-ZMO@C cathode. Sassin et al. reported the “in situ” conversion of nano-sodalite Na^+^-MnO_x_@carbon nanfoam paper (CNF) to crystalline spinel ZMO@CNF, a manganese oxide polymorph that nominally contains Zn^2+^ insertion sites [52]. ZMO@CNF cathodes were treated in a double-ended cell under electrochemical conditions and characterized in situ using XRD, SEM, energy-dispersive spectroscopy, and X-ray photoelectron spectroscopy. It is evident that the primary discharge mechanism for ZMO is the combination of proton insertion and the formation of Zn_4_(OH)_6_SO_4_·xH_2_O, despite the presence of specific Zn^2+^ insertion sites. The protons become dissociated, resulting in the dissolution of Zn_4_(OH)_6_SO_4_ when it is charged. Deng et al. introduced ZMO quantum dots into the porous carbon skeleton using in situ electrochemically induced Mn-MIL-100-derived Mn_3_O_4_ quantum dots and carbon composites [53]. The incorporation of a quantum dot structure into a ZMO-carbon composite provided a shorter ion diffusion route and increased the availability of Zn^2+^ active sites. The conductive carbon skeleton facilitated the fast electron transport. In addition, Mn–O–C bonds were formed at the interface between the ZMO quantum dots and the carbon matrix. This could effectively suppress the Jahn–Teller effect of the discharge products and the dissolution of manganese. Thus, ZMO QD@C displayed enhanced reaction kinetics, high specific capacity of 320.6 mA·h·g^−1^ at 0.1 A·g^−1^, and superior cycling stability of 1500 cycles with 86.4% capacity retention at 1 A·g^−1^. Jia et al. reported ultrafine ZnO-MnO composite nanoparticles embedded in porous carbon nanospheres as the cathode for ZIBs [54]. ZMO-MnOOH/C NSs (ZMO-MOH/C NSs) with ultrafine structure and porous conductive carbon skeleton, which were generated in situ from ZnO-MnO/C NSs, show enhanced electrical conductivity due to the shortened Zn^2+^ diffusion paths after in situ aging treatment. Moreover, The ZMO·MOH/C NSs experience a gradual enlargement during the electrochemical reaction, which reduces the drastic volume increase and guarantees the overall stability of the particle. Consequently, The ZMO-MOH/C NSs cathode offers a remarkable discharge capacity of 336.7 mA·h·g^−1^ at 0.1 A·g^−1^, and its long-term stability is remarkable, with only a 20.9% capacity loss after 1000 cycles at 1 A·g^−1^. Gao et al. used a one-step hydrothermal method to rationalize the synthesis of ZMO nanoparticles (~20 nm) on carbon nanotube (ZMO/CNTs) heterostructures to achieve stable high-rate storage of Zn^2+^ [55]. The high electrical conductivity of CNTs and minuscule ZMOs is advantageous for rapid electron transport, thus resulting in the ZMO/CNT composites having a high degree of conductivity. In addition, the combination of the pliable CNT lattice and the powerful Mn–O–C bond between ZMO and CNTs can effectively impede the irreversible structural deterioration. Accordingly, The ZMO/CNT composite exhibited a promising initial capacity of 220.3 mA·h·g^−1^ at 0.1 A·g^−1^ and maintained a high capacity retention of 97.01% after 2000 cycles at 3 A·g^−1^ (Figure 5g–i).

In summary, ZMO has been compounded with graphene, various forms of carbon materials, and CNT to enhance its electrical conductivity and, thus, improve its cycling and rate performance. Despite some progress, the complex phase transformation of ZMO during the insertion/extraction of Zn^2+^ has led to some structural degradation, making the long-term cycling performance of ZMO at high rates still unsatisfactory.

#### 3.2.2. Composite with Metal Oxides

**Figure 6:**
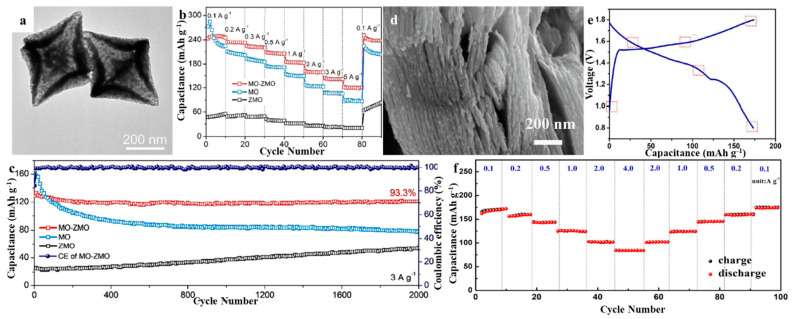
(**a**) TEM of MO-ZMO Hos. (**b**) Rate performance based on discharging curves. (**c**) Cycling performance tested at a current density of 3 A·g^−1^ of the MO-ZMO HOs, MO HOs, and ZMO HOs electrodes. Reproduced with permission from [59], Copyright 2021, Wiley-VCH. (**d**) FESEM of ZMO@Ti_3_C_2_T_x_ composite. (**e**) Galvanostatic charge−discharge curve at a current density of 0.1 A·g^−1^. (**f**) Rate capability at various current densities from 0.1 to 4 A·g^−1^. Reproduced with permission from [60], Copyright 2020, Elsevier.

Some metal oxides compounded with ZMO can improve its zinc storage capacity and improve its cyclic stability. Yang et al. prepared ZMO/Mn_2_O_3_ composites using a surfactant-assisted solvothermal method and annealed them at 600 °C for 4 h in air [56]. When used as a cathode for ZIBs, the initial discharge capacity of 82.6 mA·h·g^−1^ at 0.5 A·g^−1^ could be reached even after 300 cycles using only 1 mol·L^−1^ ZnSO_4_ as the electrolyte, and an impressive reversible capacity of 111.9 mA·h·g^−1^ could still be obtained. It also exhibited a capacity as high as 42.1 mA·h·g^−1^ when the current density increased to 3.2 A·g^−1^. The synergistic effect of ZMO and Mn_2_O_3_, the ultrafast ion transport properties, and the small self-discharge phenomenon are the primary reasons for the good cyclability and remarkable rate performance of ZMO and Mn_2_O_3_. Ma et al. reported ZMO/Mn_2_O_3_ bicomponent nanorods obtained on the basis of calcination of metal–organic skeleton-derived 2-methylimidazole zinc salt (ZIF-8)/Mn_2_O_3_ nanocomposites as cathodes for ZIBs [57]. The as-prepared ZMO/Mn_2_O_3_ demonstrated a reversible discharge capacity of 230 mA·h·g^−1^ at 0.1 A·g^−1^ after 120 cycles and a high capacity of 80 mA·h·g^−1^ at a large current density of 1 A·g^−1^. The synergistic effect of ZnMn_2_O_4_ and Mn_2_O_3_ is the main reason for its good zinc storage properties. Qin et al. used hydrothermal reaction and subsequent sintering process to prepare ZMO nanoparticles with a coating of Cu^0^-doped CuO (ZMO/CuO) [58]. The Cu^0^-doped CuO coating exhibited the synergistic effect of two-phase composites, which effectively enhanced the conductivity and charge/discharge electrochemical properties of the composites. The ZMO/CuO composite was identified as a suitable cathode material for ZIBs, exhibiting a discharge-specific capacity of 150 mA·h·g^−1^ after an activation process at 0.3 mA·g^−1^ and a capacity of 118 mA·h·g^−1^ at a current density of 2 A·g^−1^. Additionally, the Coulomb efficiency was maintained at a high level of 96% or above throughout the cycles. Zeng et al. used a two-step metal–organic backbone (MOF) template method to synthesize heterogeneous Mn_2_O_3_-ZMO hollow octahedra (MO-ZMO HOs) for the storage of zinc ions [59]. The advantageous combination of distinct chemistry and architecture enabled the MO-ZMO HO electrode to demonstrate a considerable rechargeable capacity of 247.4 mA·h·g^−1^, resulting in an impressive energy density of 305.4 W·h·kg^−1^. The MO-ZMO HO electrode exhibited improved rate performance and extended cycle life, with a capacity retention of 93.3% after 2000 cycles (Figure 6a–c). Multiple allosteric characterizations confirmed the reversible insertion and extraction reaction mechanism of Zn^2+^ in MO-ZMO HOs and verified their good structural stability.

ZMO can also be compounded with MXene to improve its cycling performance. Shi et al. investigated in depth the phase and structural evolution of spinel ZMO through in situ and ex situ studies, which are closely related to the reversible phase transition of spinel ZMO with MnO_2_ during charging and discharging, and the formation of irreversible ZnO inactive byproducts leads to capacity loss after multiple cycles [60]. They designed and synthesized Ti–Mxene (Ti_3_C_2_T_x_)-stabilized 3D assemblies of ZMO nanoparticles, in which highly conductive Ti_3_C_2_T_x_ scaffolds effectively inhibited the irreversible structural degradation and side-reactions of spinel ZMO. Thus, the ZMO@Ti_3_C_2_T_x_ composite cathode demonstrated an impressive reversible specific capacity, superior rate capability, and remarkable cyclic stability (with a capacity retention of 92.4% after 5000 cycles) (Figure 6d–f).

Despite some advancements in the combination of ZMO with metal oxides and MXenes, the extended-term cycling performance of ZMO in aqueous solutions has yet to be improved due to certain irreversible electrochemical reactions that occur during the process of Zn^2+^ insertion/extraction. Therefore, finding the right material to compound is particularly important.

### 3.3. Enlarging the Interlayer Spacing of ZMO

**Figure 7:**
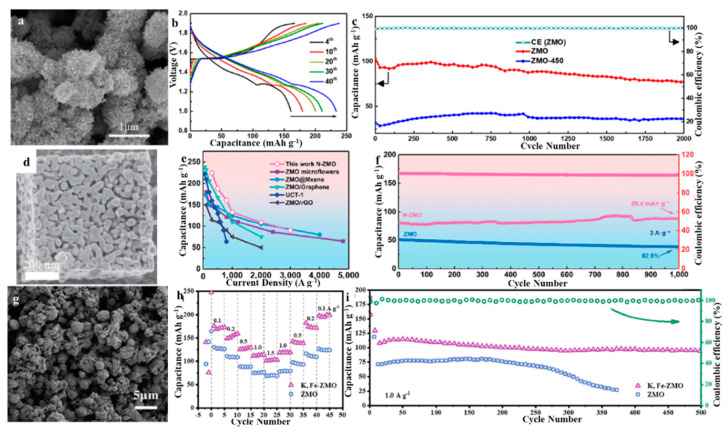
(**a**) SEM of ZMO-450. (**b**) Charge/discharge profiles of ZMO measured at 0.5 A·g^−1^ in 1 M ZnSO_4_ with 0.1 M MnSO_4_. (**c**) Cycle performance of ZMO and ZMO-450 measured at 4 A·g^−1^ in 1 M ZnSO_4_ with 0.1 M MnSO_4_ and the corresponding Coulombic efficiency of ZMO. Reproduced with permission from [61] Copyright 2021, American Chemical Society. (**d**) SEM of N-ZMO. (**e**) Discharge/charge profiles at 0.3 A·g^−1^. (**f**) Cycling performance of N-ZMO at 0.3 A·g^−1^. Reproduced with permission from [63], Copyright 2022, Springer. (**g**) SEM of K, Fe-ZMO. (**h**) Comparison of cycling performance for K, Fe-ZMO, and ZMO at 0.1 A·g^−1^. (**i**) Long-term cycle performances of K, Fe-ZMO, and ZMO. Reproduced with permission from [64], Copyright 2022, Elsevier.

ZMO is seen as a promising material for cathodes in ZIBs; however, the interfacial charge transfer resistance of divalent Zn^2+^ is typically high, thus slowing the transfer rate of Zn^2+^ at the junction between the cathode and electrolyte. Enlarging the layer spacing is considered to be an effective method to solve this challenge. Wu et al. synthesized spinel ZMO with the presence of water between the layers via a scalable low-temperature process, which remarkably overcame the problem of high resistance to interfacial charge transfer [61]. ZMO was found to have outstanding electrochemical performance when used for Zn storage, with a high capacity of 230 mA·h·g^−1^ at 0.5 A·g^−1^ and 101 mA·h·g^−1^ at 8 A·g^−1^, as well as a high specific energy/specific power of 329 W·h·kg^−1^/706 W·kg^−1^ and 134 W·h·kg^−1^/11,160 W·kg^−1^, respectively. Furthermore, it was observed to retain 75% of its capacity after 2000 cycles at 4 A·g^−1^. The ZMO-450 sample, which was created by heat posttreatment of ZMO at 450 °C and which lacked structural water, displayed a restricted discharge capacity of 45 and 15 mA·h·g^−1^ at 0.5 and 8 A·g^−1^, respectively (Figure 7a–c). Electrochemical impedance spectroscopy revealed that the presence of abundant structural water in ZMO facilitates the insertion of Zn^2+^, thus reducing the activation energy barrier and accelerating the Zn^2+^ storage interface dynamics. The Zn^2+^ participation in the charge–discharge process was highly reversible using the abundant structural water in ZMO, as characterized by XRD and X-ray photoelectron spectroscopy. Wu et al. proposed a strategy design for the preparation of nanostructured MnO/Mn_3_O_4_ materials using glucose as an intermediary, which was then converted into lattice-extended Zn_x_Mn_2_O_4_ nanoparticles via electrochemical activation [62]. The swollen structure of Zn_x_Mn_2_O_4_ can better accommodate Zn^2+^ and H^+^ ions and undergo reversible lattice expansion/contraction during charge/discharge. Accordingly, the lattice-expanded Zn_x_Mn_2_O_4_ demonstrated improved cycle performance after 2000 cycles at 1 A·g^−1^, with a capacity of 121 mA·h·g^−1^; this was significantly higher than the parent MnO_2_ (63 mA·h·g^−1^) and crystalline ZMO (58 mA·h·g^−1^).

Increasing the layer spacing of spinel ZMO is anticipated to be an effective method to create dependable electroactive materials for ZIBs, in accordance with these research findings. However, research work in this area is relatively scarce, which should be enhanced in the subsequent studies of zinc storage in ZMO.

### 3.4. Introducing Deficiencies into ZMO

The introduction of defects is considered to be a powerful technique for imparting multiple functions to materials. Defect engineering has emerged as an active strategy to improve the inherent electrochemical reactivity and structural stability of ZIBs, where defects in ZMO can improve their electrical conductivity to enhance zinc storage. Zhang et al. reported the utilization of nonstoichiometric ZMO/carbon composite as a novel inserted Zn cathode material in aqueous phase Zn(CF_3_SO_3_)_2_ electrolyte [65]. In 3 M Zn(CF_3_SO_3_)_2_ solution, 100% galvanization/zinc stripping efficiency could be achieved with long cycle stability and inhibition of Mn dissolution; spinel/carbon hybrids exhibited reversible capacity of 150 mA·g^−1^ and capacity retention of 94% over 500 cycles at a high rate of 0.5 A·g^−1^. A comprehensive suite of electrochemical measurements, XRD, Raman spectroscopy, X-ray absorption spectroscopy, Fourier-transform infrared spectroscopy, and nuclear magnetic resonance analysis revealed that the spinel structure is structurally robust with a small particle size and a high concentration of cation vacancies, facilitating efficient charge transfer and zinc insertion, resulting in remarkable electrode performance. Zhang et al. explored the kinetics of Zn^2+^ insertion/extraction in the OD-ZMO lattice by carefully extracting the oxygen anion from the ZMO cathode (OD-ZMO) [66]. Experimental and computational results showed that the oxygen defect has a well-modulated effect on the electronic conductivity, Zn^2+^ diffusion kinetics, and the energy barrier of Zn migration. The ZMO cathode, which was constructed with an oxygen-deficient composition and was protected structurally by the conductive poly (3,4-ethylenedioxythiophene), displayed a remarkable capacity of 221 mA·h·g^−1^ when tested at 0.5 mA·cm^−2^. An all-solid-state ZIBs with great flexibility was presented, exhibiting an energy density of 273.4 W·h·kg^−1^. Qiu et al. developed a new type of N-doped coupled oxygen vacancy-modulated ZMO nanotube arrays (N-ZMO NTAs) as an effective cathode for ZIBs [67]. Taking advantage of their advantageous properties such as high electrical conductivity, rapid ion diffusion, high surface area, plentiful active sites, and a secure hollow nanotube structure, N-ZMO NTAs displayed an outstanding capacity (223 mA·h·g^−1^ at 0.1 A·g^−1^), good rate capability (133.3 mA·h·g^−1^ at 4 A·g^−1^), and impressive long-term durability (92.1% after 1500 cycles). A quasi-solid-state zinc-ion battery (ZIBs) was developed using a N-ZMO NTA cathode, offering outstanding energy density (214.6 W·h·kg^−1^), remarkable power density (4 kW·kg^−1^), and impressive durability (88.6% after 1500 cycles). Huang et al. prepared mesoporous ZMO nanocages (N-ZMO) containing nitrogen doping and oxygen vacancies as high-performance cathode materials for ZIBs via defect engineering and rational structural design [63]. The presence of oxygen vacancies increased the electrical conductivity of the material, while nitrogen doping reduced the strong electrostatic force of the material, thus preserving its structural integrity. Thus, N-ZMO displayed excellent ability of Zn^2+^ storage (225.4 mA·h·g^−1^ at 0.3 A·g^−1^) with a good rate and stable cycling performance (88.4 mA·h·g^−1^ after 1000 cycles at 3 A·g^−1^). The quasi-solid-state devices were highly flexible and possess a remarkable energy density of 261.6 W·h·kg^−1^, with an impressive capacity retention of 87.5% at 3 A·g^−1^ after 1000 cycles (Figure 7d–f). Mallick et al. rationally designed and prepared defect-rich ternary spinel Zn_0.65_Ni_0.58_Mn_1.75_O_4_ with electrochemical activity, in which Mn^3+^ and Zn^2+^ were partially replaced by Ni^2+^/^3+^ [68]. The increase in ionic vacancies and tunnel size facilitated the smooth diffusion of Zn^2+^ ions. Density functional theory (DFT) calculations showed that the transformation of semiconductor non-defective ZMO into metallic ZMO ensures enhanced electronic conductivity and facilitates the storage of Zn^2+^ ions. Thus, the as-prepared Zn_0.65_Ni_0.58_Mn_1.75_O_4_ delivered a specific capacity as high as 265 mA·h·g^−1^ at 0.1 A·g^−1^ and a twofold improvement in the specific capacity after 5000 cycles. A 1 year self-discharge study exhibited that the device retained 63% of its initial performance.

Although the above research work used defective strategies to improve the Zn^2+^ storage property of ZMO cathode materials, the cycling stability of ZMO materials is limited, and achieving high cycling stability is difficult.

### 3.5. Doping Metal Ions of ZMO

The doping of heteroions with different valence states can introduce additional defects to effectively modulate the electrochemical properties. Similarly, this strategy can be used to stabilize the ZMO and increase its zinc storage capacity. Shao et al. developed double-doped ZMO (K, Fe-ZMO) as the anode material for ZIBs by modifying the crystal structure with doped Fe and low-valent K [64]. Specifically, the K, Fe-ZMO showed a high specific capacity of 221.2 mA·h·g^−1^ at 0.1 A·g^−1^ after 50 cycles and a capacity retention of 88.1% after 500 cycles at 1.0 A·g^−1^ (Figure 7g–i). DFT analysis revealed that double doping and oxygen defects could catalyze electron redistribution, resulting in increased conductivity and subsequently improved reaction kinetics and electrochemical properties of K, Fe-ZMO. The modification also reduced the formation energy, thereby effectively stabilizing the Mn-O and Fe-O bonds of K and Fe-ZMO and alleviating the dissolution of manganese. The increase in electronic conductivity and the decrease in electrostatic repulsion caused by doping and defects alike played a key role in promoting the reversible insertion/deinsertion of Zn^2+^ ions in ZMO, providing long-term cycling stability. However, relevant reports in this area are relatively scarce, which should be of interest in subsequent studies.

## 4. Conclusions and Prospects

In recent years, great progress has been made in the synthesis, characterization, electronic structure, and excellent properties of ZMO-based ZIBs. This review first briefly introduced the zinc storage mechanism of ZMO, and then reviewed the recent research progress in improving the interlayer spacing, structural stability, and diffusivity of ZMO, including the introduction of different intercalated ions, introduction of defects, design of different morphologies, and combination with other materials (Table 1). However, the development of ZMO-based ZIBs is still in its infancy, and many fundamental issues need to be urgently addressed to meet future industrial applications. Therefore, accelerating the development of high-performance ZMO-based ZIBs requires sufficient attention to the following aspects:

1. Energy storage mechanisms play an important role in guiding the design of ZMO-based materials. Many energy storage mechanisms have been proposed, but no consensus has been reached. Therefore, more high-resolution in situ characterization techniques and various electrochemical methods, as well as the development of accurate theoretical calculations, are needed to provide further evidence for the elucidation of the real storage mechanism of ZMO-based materials, which is important for the application of ZMO-based materials in ZIBs cathodes.

2. ZMO-based cathode materials can be continuously dissolved in the electrolyte solution. Therefore, an effective and novel strategy, such as incorporating additives to the electrolyte, altering the pH of the electrolyte, and forming a protective layer on the surface of the ZMO-based cathode, must be developed to prevent manganese dissolution. In aqueous solutions, the capacity of ZMO decreases mainly due to the structural transformation caused by the dissolution of the active material and the disproportionation reaction of Mn^3+^. This side reaction can be suppressed by adding Mn^2+^ salt in advance to the electrolyte. The introduction of Mn^2+^ alters the balance between the dissolution and reoxidation of Mn^2+^. It aids in the creation of a consistent, non-crystalline protective coating on the cathode electrode, thus preserving its integrity.

3. The low conductivity of ZMO-based cathode materials limits the rate capability of the ZIBs. On the basis of these issues, strategies, such as compounding with conductive carbon-based materials (e.g., CNTs, acetylene black, carbon black, and graphene), surface coating, electrolyte optimization, and combination with conductive polymers (e.g., PANI and PEDOT), can enhance the conductivity and performance of electrodes. In addition to inorganic cathode materials, organic cathode materials, which have abundant precursors and adjustable structures, may be desirable for nonaqueous and aqueous ZIBs systems in the future.

4. The successful fabrication of ZMO-based ZIBs requires the selection of suitable anodes, electrolytes, current collectors, and binders. An appropriate separator design can ensure a consistent flow of Zn^2+^ ions, while a well-made collector with a good connection to the electrode can significantly enhance the electrode’s stability. However, there are few studies focusing on functional separators and current collectors. It is essential to develop separators with excellent mechanical strength, thermal stability, and electrolyte wettability, and the production methods must be efficient and ecofriendly to satisfy the practical uses of ZIBs. In terms of current collectors, the advancement of polymer-based materials and structures that are both flexible and conductive, enabling the customization of mechanical and electrical properties, is essential for the future progress of flexible ZIBs.

In summary, the progress of ZIBs requires further systematic and scientific research. The ongoing search for and improvement of new cathode and anode materials and electrolytes are of paramount importance. Polymer electrolytes in solid or gel form offer a promising solution to various issues associated with ZIBs, such as cathode dissolution, zinc corrosion, passivation, and dendrite growth. These electrolytes have the potential to be used in the production of flexible and wearable devices in the future. ZIBs offer a number of advantages, such as affordability, ecological sustainability, and safety advantages, making them a potentially attractive option.

## Figures and Tables

**Table 1:** Comparison of the reported ZIBs based on ZMO-based materials as the cathode.

Devices (Anode//Cathode)	Voltage Window (V)	Electrolyte	Cycle Performance	Specific Capacitance	Ref
Zn foil//hollow porous ZMO	0.8–1.9	1 M ZnSO_4_ + 0.05 M MnSO_4_	59.2%, 300 cycles, 0.1 A·g^−1^	137.4 mAh·g^−1^	[40]
Zn foil//ZMO	0.1–2.1	1 M ZnSO_4_	99.26%, 25 cycles, 0.5 A·g^−1^	50.2 F·g^−1^	[41]
Zn foil//ZMO	0.6–1.9	1 M ZnSO_4_ + 0.05 M MnSO_4_	79%, 1000 cycles, 2 A·g^−1^	172 mAh·g^−1^	[42]
Zn foil//ZMO/NG	0.8–1.8	1 M ZnSO_4_ + 0.05 M MnSO_4_	97.4%, 2500 cycles, 1 A·g^−1^	221 mAh·g^−1^	[44]
Zn foil// rGO@HM-ZMO	0.8–1.8	1 M ZnSO_4_ + 0.05 M MnSO_4_	94.1%, 650 cycles, 1 A·g^−1^	146.9 mAh·g^−1^	[45]
Zn foil//ZnNi_x_Co_y_ Mn_2−x−y_O_4_@N-rGO	0.7–1.7	2 M ZnSO_4_ + 0.2 M MnSO_4_	79%, 900 cycles, 1 A·g^−1^	200.5 mAh·g^−1^	[46]
Zn foil// N-ZnMn_2_O_4-x_/VG	0.8–1.8	4 M Zn(CF_3_SO_3_)_2_	92.6%, 3000 cycles, 1 A·g^−1^	222 mAh·g^−1^	[47]
Zn foil//ZMO NDs/rGO	1.0–1.8	1 M ZnSO_4_ + 0.1 M MnSO_4_	84.1%, 400 cycles, 1 A·g^−1^	207.6 mAh·g^−1^	[48]
Zn foil//ZMO@PCPs	0.8–1.8	1 M ZnSO_4_ + 0.5 M MnSO_4_	90.3%, 2000 cycles, 1 A·g^−1^	125.6 mAh·g^−1^	[49]
Zn foil//ZMO@C	1.0–1.8	2 M ZnSO_4_ + 0.1 M MnSO_4_	-	481 mAh·g^−1^	[50]
Zn foil//Mn-d-ZMO@C	0.8–1.9	2 M ZnSO_4_ + 0.2 M MnSO_4_	84%, 2000 cycles, 0.1 A·g^−1^	194 mAh·g^−1^	[51]
Zn foil//ZMO@CNF	0.9–1.8	1 M ZnSO_4_	50%, 400 cycles, 1 C	139 mAh·g^−1^	[52]
Zn foil//ZMO QD@C	0.9–1.8	1 M ZnSO_4_	86.4%, 1500 cycles, 1 A·g^−1^	320.6 mAh·g^−1^	[53]
Zn foil// ZMO-MOH/C NSs	0.8–1.8	2 M ZnSO_4_ + 0.2 M MnSO_4_	79.1%, 1000 cycles, 1 A·g^−1^	336.7 mAh·g^−1^	[54]
Zn foil//ZMO/CNTs	0.4–1.8	1 M ZnSO_4_ + 0.1 M MnSO_4_	97.1%, 2000 cycles, 3 A·g^−1^	220.3 F·g^−1^	[55]
Zn foil//ZMO/Mn_2_O_3_	0.8–1.8	1 M ZnSO_4_	74%, 300 cycles, 0.5 A·g^−1^	151.9 mAh·g^−1^	[56]
Zn foil//ZMO/Mn_2_O_3_	0.8–1.9	2 M ZnSO_4_	79%, 120 cycles, 0.1 A·g^−1^	243.6 mAh·g^−1^	[57]
Zn foil//ZMO/CuO	0.6–1.8	2 M ZnSO_4_ + 0.1 M MnSO_4_	96%, 1000 cycles, 2 A·g^−1^	150 mAh·g^−1^	[58]
Zn//MO-ZMO HOs	0.8–1.8	2 M ZnSO_4_	93.3%, 2000 cycles, 3 A·g^−1^	247.4 mAh·g^−1^	[59]
Zn foil// ZMO@Ti_3_C_2_T_x_	0.8–1.8	2 M ZnSO_4_ + 0.1 M MnSO_4_	92.4%, 5000 cycles, 1 A·g^−1^	172.6 mAh·g^−1^	[60]
Zn foil//ZMO	1.0–1.9	1 M ZnSO_4_	75%, 2000 cycles, 4 A·g^−1^	230 mAh·g^−1^	[61]
Zn foil//ZnxMn2O4	1.0–1.9	3 M ZnSO_4_	85%, 3000 cycles, 4 A·g^−1^	170 mAh·g^−1^	[62]
Zn foil//ZMO/carbon	0.8–2.0	3M Zn(CF_3_SO_3_)_2_	94%, 500 cycles, 0.5 A·g^−1^	150 mAh·g^−1^	[65]
Zn foil//ZMO	0.8–1.9	1 M ZnSO_4_	93.8%, 300 cycles, 0.5 A·g^−1^	221 mAh·g^−1^	[66]
Zn foil//N-ZMO NTAs	0.8–1.8	2 M ZnSO_4_	92.1%, 1500 cycles, 0.5 A·g^−1^	223 mAh·g^−1^	[67]
Zn foil//N-ZMO	0.8–1.8	2 M ZnSO_4_ + 0.2 M MnSO_4_	88.4%, 1000 cycles, 3 A·g^−1^	225.4 mAh·g^−1^	[63]
Zn foil//Zn_0.65_Ni_0.58_ Mn_1.75_O_4_	0.8–2.0	3 M ZnSO_4_ + 0.1 M MnSO_4_	94%, 5000 cycles, 2 A·g^−1^	265 mAh·g^−1^	[68]
Zn foil// K, Fe-ZMO	0.7–1.7	2 M ZnSO_4_ + 0.2 M MnSO_4_	88.1%, 500 cycles, 1 A·g^−1^	221.2 mAh·g^−1^	[64]

Acronym definitions: ZnMn_2_O_4_/N-doped graphene nanocomposite (ZMO/NG), graphene-wrapped hollow ZnMn_2_O_4_ microspheres (rGO@HM-ZMO), nickel and cobalt co-substituted spinel ZnMn_2_O_4_ nanoparticles loaded onto N-doped reduced graphene oxide (ZnNi_x_Co_y_Mn_2−x−y_O_4_@N-rGO), nitrogen-modulated zinc manganite vertical graphene array (N-ZnMn_2_O_4−x_/VG), ZnMn_2_O_4_ nanodots anchored on reduced graphene oxide (ZMO NDs/rGO), ZMO nanoparticles anchored on porous carbon polyhedrons (ZMO@PCPs), double-shell ZnMn_2_O_4_ hollow microspheres embedded in 2D carbon networks (ZMO@C), carbon nanofoam paper (CNF), ZnMn_2_O_4_-MnOOH/C NSs (ZMO-MOH/C NSs), ZMO nanoparticles on carbon nanotubes (ZMO/CNTs), Mn_2_O_3_-ZnMn_2_O_4_ hollow octahedrons (MO-ZMO HOs), N-doping coupled oxygen vacancy-modulated ZMO nanotube arrays (N-ZMO NTAs), mesoporous ZnMn_2_O_4_ nanocage coupled with nitrogen doping and oxygen vacancies (N-ZMO), K and Fe double-doped ZnMn_2_O_4_ (K, Fe-ZMO).

## Data Availability

Not applicable.
